# Clinical outcomes and immune benefits of anti-epileptic drug therapy in HIV/AIDS

**DOI:** 10.1186/1471-2377-10-44

**Published:** 2010-06-17

**Authors:** Kathy Lee, Pornpun Vivithanaporn, Reed A Siemieniuk, Hartmut B Krentz, Ferdinand Maingat, M John Gill, Christopher Power

**Affiliations:** 1 Southern Alberta Clinic, Alberta Health Services, Calgary, AB, Canada; 2 Division of Neurology, Department of Medicine, University of Alberta, Edmonton, AB, Canada; 3 Department of Pharmacology, Faculty of Science, Mahidol University, Bangkok, Thailand; 4 Department of Medicine, University of Calgary, Calgary, AB, Canada; 5 Department of Microbiology and Infectious Diseases, University of Calgary, Calgary, AB, Canada

## Abstract

**Background:**

Anti-epileptic drugs (AEDs) are frequently prescribed to persons with HIV/AIDS receiving combination antiretroviral therapy (cART) although the extent of AED use and their interactions with cART are uncertain. Herein, AED usage, associated toxicities and immune consequences were investigated.

**Methods:**

HIV replication was analysed in proliferating human T cells during AED exposure. Patients receiving AEDs in a geographically-based HIV care program were assessed using clinical and laboratory variables in addition to assessing AED indication, type, and cumulative exposures.

**Results:**

Valproate suppressed proliferation *in vitro *of both HIV-infected and uninfected T cells (*p <*0.05) but AED exposures did not affect HIV production *in vitro*. Among 1345 HIV/AIDS persons in active care between 2001 and 2007, 169 individuals were exposed to AEDs for the following indications: peripheral neuropathy/neuropathic pain (60%), seizure/epilepsy (24%), mood disorder (13%) and movement disorder (2%). The most frequently prescribed AEDs were calcium channel blockers (gabapentin/pregabalin), followed by sodium channel blockers (phenytoin, carbamazepine, lamotrigine) and valproate. In a nested cohort of 55 AED-treated patients receiving cART and aviremic, chronic exposure to sodium and calcium channel blocking AEDs was associated with increased CD4+ T cell levels (*p <*0.05) with no change in CD8+ T cell levels over 12 months from the beginning of AED therapy.

**Conclusions:**

AEDs were prescribed for multiple indications without major adverse effects in this population but immune status in patients receiving sodium or calcium channel blocking drugs was improved.

## Background

Anti-epileptic drugs (AEDs) are frequently used as adjunct therapies for several conditions aside from epilepsy and seizures including movement disorders, mood disorders and neuropathic pain [1,2]. The individual choice of AED is usually made based on the specific indication and potential drug side-effect profile such as hepatic or renal dysfunction, leukopenia, and the patient's co-morbidities as well as concurrent treatments. Nevertheless, monitoring blood AED levels can reduce the incidence of specific AED side-effects. Co-morbid diseases often complicate the use of AEDs, in large part because of their consequences such as organ failure and/or neuropsychiatric effects [3,4]. Human immunodeficiency virus (HIV) infection is associated with a higher prevalence of neuropathic pain (25-50%) [5], seizures/epilepsy (3-6%) [6,7], and mood disorders [8] than within the general population and often require AED treatment(s) [9-11]. However, the prescription of AEDs in the context of HIV infection, especially in the acquired immunodeficiency disease syndrome (AIDS) phase, can present substantial clinical challenges, given the accompanying risks of hepatic or renal failure together with the increasingly complex range of antiretroviral therapies prescribed for HIV/AIDS. Indeed, some components of combination antiretroviral therapy (cART) such as protease inhibitors often pose serious risks in terms of drug interactions, occasionally with life threatening consequences in individuals who already have multi-organ diseases [12,13]. Despite these concerns, AEDs continue to be used widely in HIV/AIDS patients receiving cART, albeit with uncertainty regarding potential adverse consequences. The full spectrum of AED use in HIV-infected patients remains unknown, nor is the risk of adverse effects accompanying AED use. Moreover, clinicians caring for patients who receive both AEDs and concurrent antiretroviral drugs face clinical dilemmas arising from the potential interactions between both classes of drugs. Little is known about the impact AEDs on immunologic and virologic markers during HIV infection although *in vitro *studies suggest that some AEDs (valproate) might enhance viral replication while the *in vivo *effects remain less certain [14].

Given these complex circumstances, the working hypothesis was: as AEDs are frequently prescribed for protracted periods for a variety of conditions in HIV/AIDS patients, AEDs might exert substantial effects on virologic, immunologic and clinical outcomes. Viremic status provides a robust indicator of control of HIV infection, which can be used to monitor potentially adverse effects of other interventions such as AEDs initiation, as assessed in the present studies. Herein, we investigated the extent and impact of AED use among aviremic and viremic persons with HIV/AIDS attending a regional HIV program as well as the *in vitro *effects of frequently used AEDs on T cell proliferation and HIV replication.

## Methods

### Laboratory studies

Primary human peripheral blood lymphocytes (PBLs) were purified from healthy HIV seronegative subjects' blood with Histopaque (Sigma) and maintained in RPMI 1640 medium with 15% FBS with phytohemagglutinin-P (PHA-P) stimulation for 3 days, followed by hIL-2 stimulation and/or an anti-hCD3 monoclonal antibody (eBioscience, San Diego, CA) for the duration of the experiment [15]. HIV-1 SF162 stocks were used to infect PBLs at day 3 post-isolation of the cells and then treated with gabapentin, valproate or phenytoin (20, 75 and 15 μg/ml, respectively, Sigma) for the duration of the experiment. T cell proliferation was assessed at days 2 and 4 post-infection by CellTrace™ CFSE Cell Proliferation Kit (Molecular Probes, Eugene, OR) and FACS analyses together with reverse transcriptase activity [16].

### Clinical Investigations

The Southern Alberta Clinic (SAC) is a multidisciplinary, geographically-based clinical program, which provides care to all HIV seropositive patients in southern Alberta, Canada [17]. Participation in this study was voluntary and an informed consent was approved by the University of Calgary Ethics Committee. Clinical and laboratory variables were assessed every three to four months, which included complete blood counts, electrolytes, hepatic and renal function tests, blood CD4+/CD8+ T cell levels, plasma viral loads and serum AED levels, when indicated. The principal objective at SAC of cART treatment is to suppress virus in blood to undetectable levels (≤1.6 log_10 _copies/ml). SAC has access to all contemporary antiretroviral drugs approved in Canada.

All adult patients receiving AEDs were identified within the clinic database (Jan 01/2001-May 01/2007), an in-house computerized database containing all relevant patient characteristics dating back to 1985, which is updated with each patient visit to the clinic. Clinical aspects, laboratory findings, demographic features and AED usage were analyzed. Liver function tests (LFTs) (alkaline phosphatase, alanine aminotransferase (ALT), aspartate aminotransferase (AST), total bilirubin) were recorded for every AED-treated patient during AED therapy. The severity of hepatotoxicity was graded based on the guideline of AIDS clinical trial group (ACTG) [18] and the total number of abnormal LFTs was recorded. Cumulative AED dosing was calculated for all patients; cumulative dose (g) was the summation of the days that patients received AEDs multiplied by the dose and frequency to the date of AED being stopped or to the end of the follow up period (May 01/2008).

AED-treated patients were stratified into two groups based on virologic status: AED-receiving patients were defined as "aviremic" if they had been receiving cART for at least one month with an undetectable plasma viral (aviremic) load (≤ 1.6 log_10 _copies/ml) prior to the introduction of an AED and remained on the same cART regimen for the full duration of AED exposure/therapy and had no episodic of protracted virologic failure (>2 log_10 _copies/ml) during the period of concomitant exposure to cART and AED; patients were defined as "viremic" if they had detectable viral loads, because they were unable to maintain sustained adherence to drug therapies or had not received antiretroviral therapy prior to AED treatment. The viral load, CD4+ and CD8+ T cell levels prior to initiating AED therapy (baseline) were compared with corresponding values at 6 and 12 month follow-up visits. Patients were not included in this sub-analysis if they did not have viral loads or CD4+ T cell levels prior to the initiation of AED therapy or received AED therapy less than 6 months.

### Statistical analysis

*In vitro *data were tested by one-way ANOVA with *post-hoc *Tukey-Kramer. Demographic and clinical variables were analyzed by the Kruskal-Wallis and Chi-square tests for non-parametric continuous and categorical variables, respectively. The levels of CD4+ and CD8+ T cells were tested by Friedman test for non-parametric repeated measures ANOVA with Dunn's multiple comparisons test as a *post hoc *analysis. The level of significance was defined as *p *less than 0.05 for all tests.

## Results

### Cell culture studies

**Figure 1:**
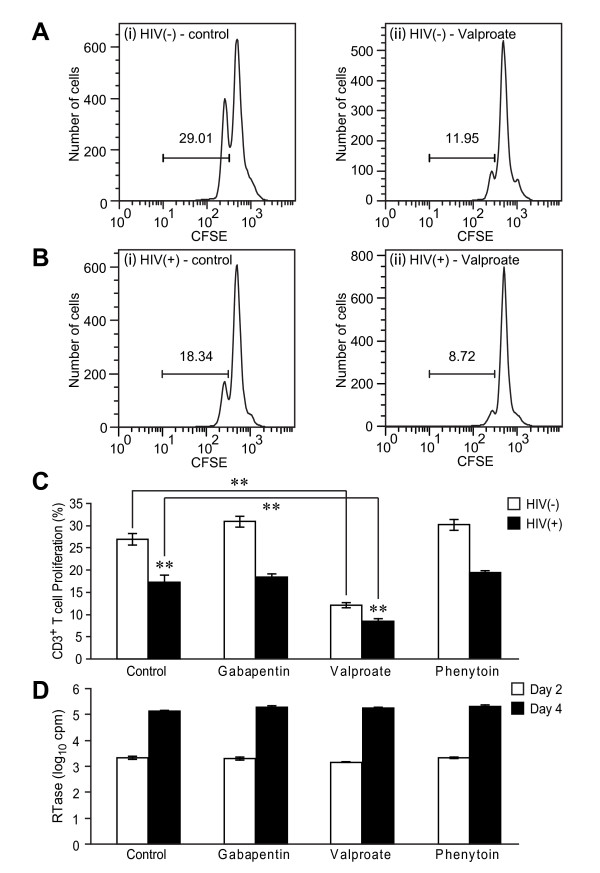
***In vitro *effects of AEDs on T cell proliferation and viral replication**. (A) and (B) FACS analysis of cultured CD3+ T cells following mock (HIV (-)) and HIV (HIV (+)) infection in the (i) absence or (ii) presence of valproate treatment (75 μg/ml) showing reduced proliferation in valproate-treated cultures at day 2 post-infection. (C) Valproate exerted a suppressive effect on T cell proliferation with and without HIV infection in contrast to the other AEDs. (D) None of the AEDs affected HIV replication in CD3+ T cells, measured as reverse transcriptase activity in culture supernatants collected at day 2 and 4 post-infection. Data represent mean ± SEM. (Tukey-Kramer *post hoc *test, **p < 0.01)

**Figure 2:**
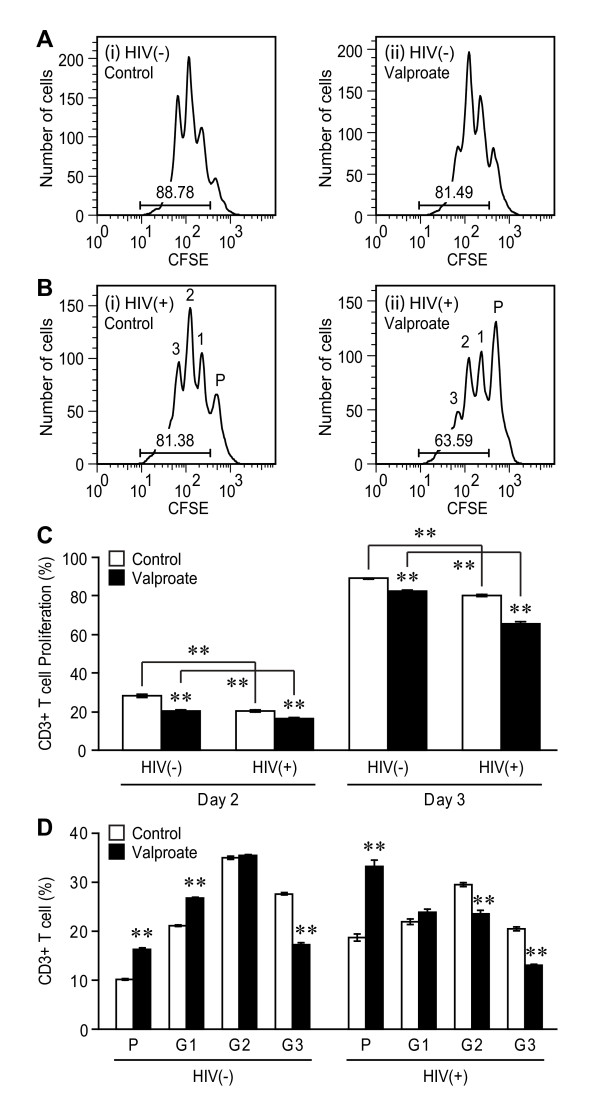
***In vitro *effects of Valproate on T cell proliferation and viral replication at day 3 post-infection**. (A) and (B) FACS analysis of cultured CD3+ T cells following mock (HIV (-)) and HIV (HIV(+)) infection in the (i) absence or (ii) presence of valproate treatment (75 μg/ml) showing reduced proliferation in valproate-treated cultures at day 3 post-infection. In the absence of valproate, most T cells divided one to three times while in the presence of valproate, the majority of T cells remained in a parent generation (P). (C) Exposure to valproate also reduced T cell proliferation with and without HIV infection at day 3 post-infection. (D) Exposure to valproate skewed the proliferation pattern of uninfected (HIV(-)) and HIV-infected (HIV(+)) PBLs. There were more parent generation (P) and less generation 3 (G3) lymphocytes in valproate-treated groups. Data represent mean ± SEM. (Tukey-Kramer *post hoc *test, **p < 0.01)

Previous studies have reported that exposure to different AEDs influenced both HIV replication and leukocyte viability or proliferation [14,19-22]. To investigate the comparative effects of commonly used AEDs, human primary blood lymphocytes (PBLs) with and without concurrent HIV-1 infection were treated with gabapentin, valproate or phenytoin at therapeutic concentrations. These studies revealed that HIV infection suppressed T cell proliferation *in vitro *(Figure 1B and 1C) compared with uninfected (Figure 1A and 1C) cell cultures at day 2 post-infection in cells, as expected. However, valproate exposure to PBLs suppressed T cell proliferation in both HIV-infected and uninfected cultures in comparison with untreated cultures (Figure 1C). Conversely, gabapentin and phenytoin did not influence T cell proliferation *in vitro *(Figure 1C). None of the present AEDs exerted effects on viral production in terms of reverse transcriptase activity in supernatants from PBLs relative to untreated HIV-infected cultures (Figure 1D). A sub-analysis of valproate exposure on T cell proliferation at day 3 post infection showed that valproate not only reduced but also altered proliferation pattern (Figure 2A-C). In uninfected lymphocyte cultures, the valproate-treated cells displayed higher parent generation (P) and generation 1 (G1) and lower generation 3 (G3) levels than the untreated group. The majority of cells in HIV-infected PBLs with valproate exposure remained undivided (P generation) while HIV-infected PBLs divided two times in the absence of valproate (Figure 2B and 2D). Similar effects were observed at day 4 post-infections (data not shown). These studies highlighted the potential impact of valproate on T cell function while also indicating a relative lack of effect on viral replication mediated by any of the AEDs on proliferating T cells, prompting us to analyse the clinical effects of these same AEDs.

### Demographic and clinical features of AED-exposed persons

169 persons (12.6%) were exposed to AED therapy among all HIV/AIDS patients (n = 1345) actively receiving care at SAC during the study period in the current cohort. Comparison of demographic and clinical features of AED-treated viremic and aviremic (stable cART regimen) patients disclosed no differences in age, mortality, AIDS status, gender, ethnicity, HIV risk factor, duration of HIV infection and CD4+ T cell nadir and viral burden at the time of first diagnosis although CD4+ T cell levels were significantly higher at the time of HIV diagnosis in the viremic group (Table 1). Collectively, these findings implied that these two groups shared similar clinical features.

**Table 1:** Demographic and clinical characteristics of HIV/AIDS patients with antiepileptic drug (AED) exposure.

	Viremic	Aviremic	*p *value
N	114	55	
Median age (IQ range)	46.2 (40.0-52.0)	49.2 (42.1-56.2)	NS
Median age at HIV diagnosis (IQ range)	33.8 (27.0-41.95)	36.0 (27.4-44.9)	NS
Male gender	84 (73.7%)	44 (80.0%)	NS
Ethnicity			NS
Caucasian	90 (78.9%)	43 (78.2%)	
Aboriginal/Inuit/Metis	7 (6.1%)	3 (5.5%)	
Black	7 (6.1%)	3 (5.5%)	
Others	10 (8.9%)	6 (10.9%)	
HIV risk factor			NS
Homosexual/Bisexual	51 (44.7%)	26 (47.3%)	
IVDU	36 (31.6%)	13 (23.6%)	
Others	27 (23.7%)	16 (29.1%)	
HCV	39 (34.2%)	12 (21.8%)	NS
Median duration of HIV infection (years) (IQ range)	11.7 (7.5-16.6)	12.6 (8.9-16.7)	NS
Median baseline CD4^+ ^T cell (cells/mm^3^) (IQ range)	371 (185-583)	241 (104-457)	<0.05
Median nadir CD4^+ ^T cell (cells/mm^3^) (IQ range)	120 (26-196)	125 (32-222)	NS
Median baseline CD8^+ ^T cell (cells/mm^3^) (IQ range)	784 (557-1134)	726 (515-1120)	NS
Median baseline log_10 _viral load (copies/ml) (IQ range)	4.5 (3.5-5.1)	4.3 (3.3-5.0)	NS
Late presenter (%)	34 (29.8%)	22 (40.0%)	NS
AIDS-defining illness	50 (43.9%)	24 (43.6%)	NS
Mortality (%)	23 (20.2%)	8 (14.5%)	NS

IQ range: Interquartile range

### AED indication, type and cumulative exposure

**Figure 3:**
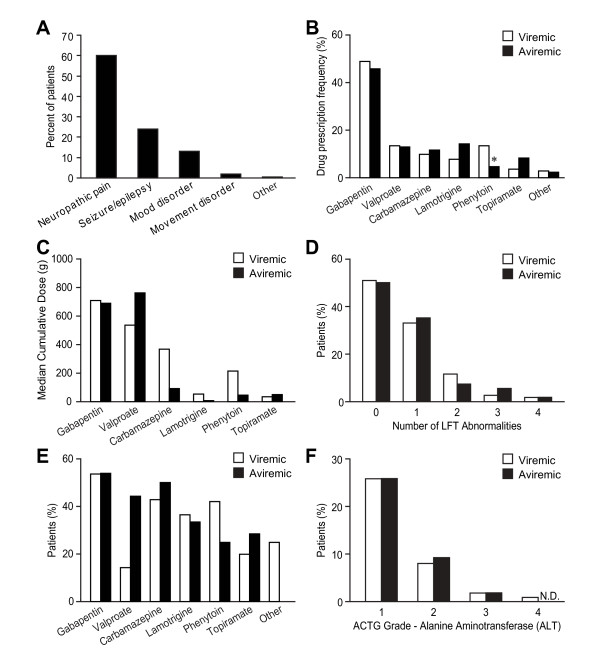
**Indication, frequency, cumulative exposure and liver toxicity of AEDs**. (A) Neuropathic pain represented the most common indication for AED treatment although seizures/epilepsy, mood and movement disorders were other reasons for prescribing AEDs. (B) A similar profile of AED prescription was observed in viremic (open box) or aviremic (filled box) patients. Gabapentin was the most frequently prescribed AED. The use of phenytoin was significantly lower in patients with stable ART regimens. (Chi-square test, *p < 0.05). (C) Cumulative AED dosing for patients receiving AEDs disclosed that gabapentin and valproate showed the highest levels of cumulative drug exposures. Among aviremic patients, the profile of cumulative dosing was similar to patients with detectable viral loads. (D) The frequency of abnormal liver function tests (LFTs) in aviremic patients with concurrent use of AED did not differ from AED-treated viremic persons. (E) The risks of abnormal LFTs in patients receiving gabapentin, carbamazepine, lamotrigine and topiramate were similar in viremic or aviremic patients while the risk of abnormal LFTs were three-times higher in aviremic patients receiving valproate. (F) Severity of hepatotoxicity was categorized based on AIDS clinical trial group (ACTG) guidelines. AED-treated viremic or aviremic patients displayed a similar profile of hepatotoxicity in the levels of alanine aminotransferase (ALT). (N.D.: non detectable)

The indications for AED therapy in the present cohort of patients were identified as peripheral neuropathy/neuropathic pain, seizure/epilepsy, mood disorders, movement disorders and other (headache) (Figure 3A). The AEDs that were prescribed in the included gabapentin/pregabalin, valproate, carbamazepine, lamotrigine, phenytoin, topiramate and others (phenobarbital, levetiracetam and primidone) (Figure 3B). Gabapentin was the most frequently prescribed AED in patients who were viremic or aviremic. The use of other AEDs except phenytoin was also similar in both groups. Multiple AEDs were prescribed in a subset of patients including 2 AEDs (13.3%) and 3 or more AEDs (9.0%). There were 143 of 169 patients who were cART-experienced with median cART exposure time of 75 months (IQR 33.3-116.5). As some patients required AED therapy for lengthy periods, we analysed the median cumulative exposures of the major AEDs during the study period. Among all AED-treated patients, the median total AED cumulative lifetime exposures (g) were: gabapentin, 699; valproate, 574; carbamazepine, 282; lamotrigine, 18; phenytoin, 195; and topiramate, 41. Aviremic patients showed a lower median total AED cumulative lifetime doses of carbamazepine, lamotrigine and phenytoin than viremic patients (Figure 3C). These findings underscored the variation in dosing for different AEDs while also highlighting the substantial exposure to AED therapies within the present cohort.

### Liver function tests and AED toxicity

The potential for metabolic interactions between AEDs and different cART regimens is substantial, particularly in terms of ensuing hepatic dysfunction [23]. To address this issue, the frequency and severity of abnormal laboratory tests was investigated among viremic patients as well as those who were aviremic. Both groups showed a similar profile of abnormalities in liver function tests (LFTs) with no abnormalities in half of patients and one abnormal LFT in one-third of patients (Figure 3D). Of the patients who had ≥3 or more LFT abnormalities 8 out of 9 patients received gabapentin. In addition, 54% of patients with gabapentin use experienced at least one abnormal LFT during AED therapy (Figure 3E). Interestingly, aviremic patients with concurrent use of valproate and stable cART regimens experienced a 3 fold higher risk of LFT abnormalities (Figure 3D). The most frequent abnormal LFT was elevated ALT (36.7%), followed by elevated alkaline phosphatase (16.9%), hyperbilirubinemia (9.6%) and elevated AST (9.0%). Most of abnormal LFTs were mild and were categorized as grade 1 hepatotoxicity in both groups except hyperbilirubinemia (Figure 3E and Additional file 1 Figure S1A-C). Only one-third of patients in both groups were experienced one additional LFT abnormality and approximately 10% of patients displayed two or more additional LFT abnormalities (Additional file 1 Figure S1D). Of overall importance, no events of overt hepatic or virologic failure were identified within these AED-treated patient groups regardless of clinical grouping during the study period. Similarly, other organ failures, hospitalization or death due to AED-mediated adverse effects were not observed in this cohort.

### Impact of AED therapy on infection status

**Figure 4:**
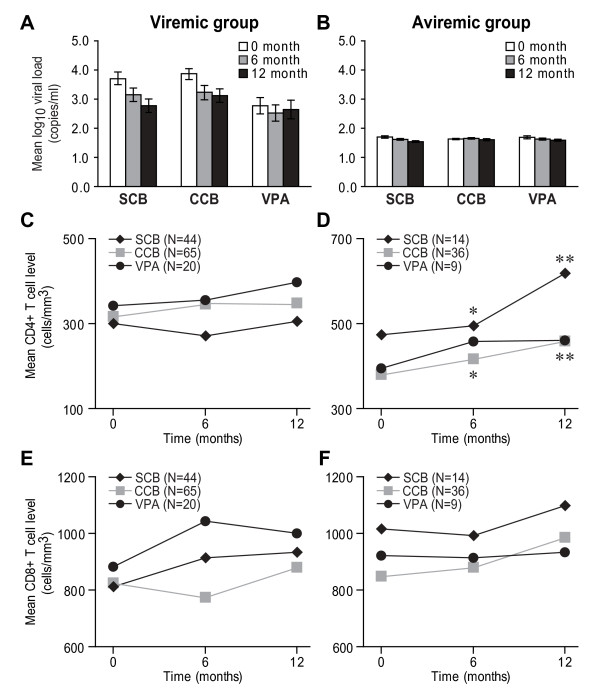
**AEDs and their effects on immune parameters**. (A) In viremic or ART-naïve patients, mean log_10 _viral load in patients treated with sodium channel blockers (SCB: carbamazepine, lamotrigine and phenytoin; N = 44), calcium channel blockers (CCB: gabapentin, pregabalin; N = 65) and valproate (N = 20) were higher than the detection limit at all time points. (B) In all AED-treated aviremic patients, mean log_10 _viral loads were maintained at or near detection levels throughout the 12 month follow-up period. (C) Mean blood CD4+ T cell levels were constant over the 12 months in viremic patients. (D) In contrast, aviremic patients receiving SCB (N = 14) and CCB (N = 36) cART had higher CD4+ T cell levels at 6 and 12 months compared to the baseline while there was no change in CD4+ T cell levels in patients treated with valproate. (E and F) AED therapies did not affect blood CD8+ T cell levels in both patient groups. (Friedman test with Dunn's multiple comparison *post hoc *test; *p < 0.05)

Previous studies suggest that AEDs might exert differential effects on viral replication and perhaps also affect lymphocyte activation state or phenotype [14,24]. To assess these effects *in vivo*, AEDs were classified based on their putative mechanisms of action within the cART and AED-exposed aviremic group including calcium channel blockers (CCB: gabapentin/pregabalin) (55.4%), sodium channel blockers (SCB: phenytoin, carbamazepine, lamotrigine) (21.5%), valproate (13.9%), and Others (topiramate, levetiracetam) (9.2%). Among the 55 patients in this group, 8 were treated with more than one AED at different times during the study period. Viremic patients or ARV-naïve patients had detectable viral loads (>2 log_10 _copies/ml) (Figure 4A). Mean plasma viral (log_10_) loads were maintained at or near detection levels in aviremic group during the 12 months of treatment irrespective of whatever AED prescribed to the patients (Figure 4B). Aviremic patients receiving CCBs, SCBs or valproate showed similar demographic profiles and there was no difference between the baseline CD4+ T cell level (0 month) (Figure 4D). Over the 12 month follow-up period beginning at the initiation of AED therapy, mean blood CD4+ T cell levels in viremic patients did not change in AED treatment group (Figure 4C). In contrast, mean blood CD4+ T cell levels at 6 and 12 follow-up months increased significantly among aviremic patients treated with SCBs or CCBs but not in patients treated with valproate (Figure 4D). Mean blood CD8+ T cell levels remained unchanged during the 12 follow-up months in both patient groups with all AED therapies (Figure 4E-F). These findings implied that AED exposure had little effect on plasma viral load levels and blood CD8+ T cell levels but there was a contemporaneous increase in blood CD4+ T cell levels in aviremic patients treated with cART and calcium or sodium channel blocking agents.

## Discussion

The present study provides the first longitudinal analysis of cumulative AED use in patients with serious systemic comorbidities and at high risk of drug interactions. Indeed, AEDs were prescribed for multiple indications with limited side-effects in this broadly representative regional population of HIV-infected persons. Despite frequent use and high cumulative doses of AEDs, no events of virologic or organ failure, attributable to AED use were recorded. Drugs within all of the AED classes were prescribed herein but sodium and calcium channel blocking AEDs were the most commonly observed with associated benefits in terms of a rise in CD4+ T cell levels over time while valproate was not associated with improvement in systemic immunity. The frequent use (>10%) of AEDs in HIV/AIDS patients also underscored the burden of neuropsychiatric diseases within this patient population yet provides assurance that AEDs can be used safely among patients receiving cART without serious adverse consequences.

Due to the numerous potential interactions between AEDs and cART in terms of hepatic metabolism, neuropsychiatric and renal side-effects, the relative paucity of adverse events herein was encouraging. In fact, minimal changes in AEDs regimens were required, as dictated by measured AED blood levels. In some instances, the AED dosages were increased to compensate for increased metabolism to achieve therapeutic AED blood levels. Although 1/3 of patients exhibited one abnormal LFT, the severities of abnormal LFTs were mild. This relative lack of undesirable interactions might have been due to regular clinical monitoring of patients with repeated AED blood levels when available (valproate, carbamazepine, phenytoin) together with the frequent use of gabapentin/pregabalin, which are excreted renally. Importantly, previous studies suggest that AEDs including gabapentin and lamotrigine were well tolerated when prescribed to patients with HIV/AIDS [11,25,26]. Nonetheless, the risk of abnormal LFTs is considerable within this group of patients; several explanations for these abnormalities lie in the demographics of the cohort including the comparatively high risk of hepatitis virus infection, substance abuse and other concomitant medical issues accompanying immune suppression.

In the present study, several patients were receiving valproate; of interest, earlier studies suggested that valproate increased HIV replication *in vitro *although the mechanism remains uncertain but this effect was thought to enhance clinical clearance of the virus from tissue reservoirs, eventually improving clinical outcomes [24]. However, the present experimental studies indicated that valproate, phenytoin and gabapentin had no effects on viral replication on T cells infected with a CCR5-dependent strain of HIV-1, similar to a previous study [14]. Conversely, valproate suppressed T cell proliferation *in vitro *regardless of the presence or absence of concomitant HIV infection, suggesting that valproate influenced the ability of T cells to divide efficiently and further analysis on the effects of antiepileptic drugs on T cell subpopulation is of interest. Indeed, valproate has recently been shown to affect proliferation of malignant cells [27,28] but had no *in vivo *effects on viral replication or CD4+ T cell levels [22,29-31]. However, the current studies showed that concurrent use of calcium (gabapentin, pregabalin) and sodium (carbamazepine, phenytoin, lamotrigine) channel blocking drugs with stable cART regimens, which maintained aviremia, exerted a benefit in terms of increased median CD4+ T cell levels in blood over a 12 month period. Several potential explanations underlie this observation including blocking cation channels and thus stabilizing lymphocyte membrane potentials and/or suppressing intracellular death signalling pathways, thereby, preventing leukocyte depletion. Alternatively, the rise in CD4+ T cell levels might reflect greater patient adherence to cART regimens because they are experiencing a better quality of life due to AED usage. Whatever the explanation for this finding, it warrants further investigation because it might provide insight into additive benefits for the treatment of HIV/AIDS.

## Conclusion

This report is the first to assess cumulative exposure of multiple AEDs in any population over time. Despite high cumulative doses of different AEDs, our study showed that the use of several AEDs in HIV-infected patients receiving cART was comparatively safe and might be beneficial to immune status. These findings are clinically relevant because AEDs are widely prescribed in HIV-infected patients with various neuropsychiatric syndromes, as well as in the general population. In summary, this is the first analysis of AED use and effects in HIV/AIDS patients closely monitored in a clinical setting, but also raised interesting questions to be explored in the future regarding immune benefits of AEDs and their underlying mechanisms.

## Abbreviations

ACTG: AIDS clinical trial group; AED: anti-epileptic drug; AIDS: acquired immune deficiency syndrome; ALT: alanine aminotransferase; ARV: antiretroviral drug; AST: aspartate aminotransferase; cART: combination antiretroviral therapy; CCB: calcium channel blocker; CFSE: carboxyfluorescein succinimidyl ester; LFT: liver function test; HIV: human immunodeficiency virus; IQR: interquartile range; PBL: peripheral blood lymphocyte; PHA-P: phytohemagglutinin-P; SAC: Southern Alberta Clinic; SCB: sodium channel blocker

## Competing interests

The authors declare that they have no competing interests.

## Authors' contributions

KL participated in the development of study concept, acquired and performed analysis of clinical data. PV carried out the *in vitro *studies, performed statistical analysis and data interpretation together with drafted and revised the manuscript content. RS and HBK took part in acquisition and analysis of clinical data. FM participated in the *in vitro *studies and data analysis. MJG was involved in study concept and design, data analysis and manuscript drafting. CP participated in study concept and design, obtained funding, as well as drafted and revised the manuscript. All the authors have read and approved the final version of the manuscript.

## Pre-publication history

The pre-publication history for this paper can be accessed here:

http://www.biomedcentral.com/1471-2377/10/44/prepub

## Supplementary Material

Additional file 1 **Figure S1. Liver toxicity of AEDs**. (A) and (B) Based on ACTG guidelines, aviremic patients with concurrent AED use had similar aspartate aminotransferase (AST) and alkaline phosphatise abnormalities to viremic patients (3A and 3B). In contrast, aviremic patients showed a trend toward lower hyperbilirubinemia (3E). Both aviremic and viremic patients displayed similar profile of additional LFT abnormalities after the initiation of AEDs (Figure 3D). Click here for file

## References

[B1] Ettinger AB Argoff CE Use of antiepileptic drugs for nonepileptic conditions: psychiatric disorders and chronic pain Neurotherapeutics 2007 4 1 75 83 10.1016/j.nurt.2006.10.003 17199018 PMC7479709

[B2] Rogawski MA Loscher W The neurobiology of antiepileptic drugs for the treatment of nonepileptic conditions Nature medicine 2004 10 7 685 692 10.1038/nm1074 15229516

[B3] Kennedy GM Lhatoo SD CNS adverse events associated with antiepileptic drugs CNS Drugs 2008 22 9 739 760 10.2165/00023210-200822090-00003 18698874

[B4] Asconape JJ Some common issues in the use of antiepileptic drugs Semin Neurol 2002 22 1 27 39 10.1055/s-2002-33046 12170391

[B5] Verma S Estanislao L Simpson D HIV-associated neuropathic pain: epidemiology, pathophysiology and management CNS drugs 2005 19 4 325 334 10.2165/00023210-200519040-00005 15813646

[B6] Kellinghaus C Engbring C Kovac S Moddel G Boesebeck F Fischera M Anneken K Klonne K Reichelt D Evers S Frequency of seizures and epilepsy in neurological HIV-infected patients Seizure 2008 17 1 27 33 10.1016/j.seizure.2007.05.017 17618132

[B7] Pascual-Sedano B Iranzo A Marti-Fabregas J Domingo P Escartin A Fuster M Barrio JL Sambeat MA Prospective study of new-onset seizures in patients with human immunodeficiency virus infection: etiologic and clinical aspects Archives of neurology 1999 56 5 609 612 10.1001/archneur.56.5.609 10328257

[B8] Cruess DG Evans DL Repetto MJ Gettes D Douglas SD Petitto JM Prevalence, diagnosis, and pharmacological treatment of mood disorders in HIV disease Biological psychiatry 2003 54 3 307 316 10.1016/S0006-3223(03)00318-4 12893106

[B9] Hahn K Arendt G Braun JS von Giesen HJ Husstedt IW Maschke M Straube ME Schielke E A placebo-controlled trial of gabapentin for painful HIV-associated sensory neuropathies Journal of neurology 2004 251 10 1260 1266 10.1007/s00415-004-0529-6 15503108

[B10] Halman MH Worth JL Sanders KM Renshaw PF Murray GB Anticonvulsant use in the treatment of manic syndromes in patients with HIV-1 infection The Journal of neuropsychiatry and clinical neurosciences 1993 5 4 430 434 8286943 10.1176/jnp.5.4.430

[B11] Simpson DM McArthur JC Olney R Clifford D So Y Ross D Baird BJ Barrett P Hammer AE Lamotrigine for HIV-associated painful sensory neuropathies: a placebo-controlled trial Neurology 2003 60 9 1508 1514 12743240 10.1212/01.wnl.0000063304.88470.d9

[B12] Romanelli F Jennings HR Nath A Ryan M Berger J Therapeutic dilemma: the use of anticonvulsants in HIV-positive individuals Neurology 2000 54 7 1404 1407 10751246 10.1212/wnl.54.7.1404

[B13] Liedtke MD Lockhart SM Rathbun RC Anticonvulsant and antiretroviral interactions The Annals of pharmacotherapy 2004 38 3 482 489 10.1345/aph.1D309 14970370

[B14] Robinson B Turchan J Anderson C Chauhan A Nath A Modulation of human immunodeficiency virus infection by anticonvulsant drugs Journal of neurovirology 2006 12 1 1 4 10.1080/13550280500516278 16595368

[B15] Power C McArthur JC Johnson RT Griffin DE Glass JD Dewey R Chesebro B Distinct HIV-1 env sequences are associated with neurotropism and neurovirulence Current topics in microbiology and immunology 1995 202 89 104 7587373 10.1007/978-3-642-79657-9_7

[B16] Johnston JB Jiang Y van Marle G Mayne MB Ni W Holden J McArthur JC Power C Lentivirus infection in the brain induces matrix metalloproteinase expression: role of envelope diversity Journal of virology 2000 74 16 7211 7220 10.1128/JVI.74.16.7211-7220.2000 10906175 PMC112242

[B17] Pandya R Krentz HB Gill MJ Power C HIV-related neurological syndromes reduce health-related quality of life The Canadian journal of neurological sciences 2005 32 2 201 204 16018155 10.1017/s0317167100003978

[B18] Ogedegbe AO Sulkowski MS Antiretroviral-associated liver injury Clinics in liver disease 2003 7 2 475 499 10.1016/S1089-3261(03)00023-0 12879995

[B19] Cloyd MW Lynn WS Ramsey K Baron S Inhibition of human immunodeficiency virus (HIV-1) infection by diphenylhydantoin (dilantin) implicates role of cellular calcium in virus life cycle Virology 1989 173 2 581 590 10.1016/0042-6822(89)90569-2 2574518

[B20] Maggi JD Halman MH The effect of divalproex sodium on viral load: a retrospective review of HIV-positive patients with manic syndromes Canadian journal of psychiatry 2001 46 4 359 362 10.1177/070674370104600409 11387794

[B21] Maggi JD Halman MH The effect of divalproex sodium on HIV replication in vivo The Journal of neuropsychiatry and clinical neurosciences 2007 19 3 326 330 17827419 10.1176/jnp.2007.19.3.326

[B22] Siliciano JD Lai J Callender M Pitt E Zhang H Margolick JB Gallant JE Cofrancesco J Jr Moore RD Gange SJ Stability of the latent reservoir for HIV-1 in patients receiving valproic acid The Journal of infectious diseases 2007 195 6 833 836 10.1086/511823 17299713

[B23] Romanelli F Pomeroy C Concurrent use of antiretrovirals and anticonvulsants in human immunodeficiency virus (HIV) seropositive patients Current pharmaceutical design 2003 9 18 1433 1439 10.2174/1381612033454676 12769723

[B24] Lehrman G Hogue IB Palmer S Jennings C Spina CA Wiegand A Landay AL Coombs RW Richman DD Mellors JW Depletion of latent HIV-1 infection in vivo: a proof-of-concept study Lancet 2005 366 9485 549 555 10.1016/S0140-6736(05)67098-5 16099290 PMC1894952

[B25] La Spina I Porazzi D Maggiolo F Bottura P Suter F Gabapentin in painful HIV-related neuropathy: a report of 19 patients, preliminary observations Eur J Neurol 2001 8 1 71 75 10.1046/j.1468-1331.2001.00157.x 11509084

[B26] Archin NM Eron JJ Palmer S Hartmann-Duff A Martinson JA Wiegand A Bandarenko N Schmitz JL Bosch RJ Landay AL Valproic acid without intensified antiviral therapy has limited impact on persistent HIV infection of resting CD4+ T cells AIDS (London, England) 2008 22 10 1131 1135 18525258 10.1097/QAD.0b013e3282fd6df4 PMC3863687

[B27] Hrzenjak A Moinfar F Kremser ML Strohmeier B Staber PB Zatloukal K Denk H Valproate inhibition of histone deacetylase 2 affects differentiation and decreases proliferation of endometrial stromal sarcoma cells Molecular cancer therapeutics 2006 5 9 2203 2210 10.1158/1535-7163.MCT-05-0480 16985053

[B28] Li XN Shu Q Su JM Perlaky L Blaney SM Lau CC Valproic acid induces growth arrest, apoptosis, and senescence in medulloblastomas by increasing histone hyperacetylation and regulating expression of p21Cip1, CDK4, and CMYC Molecular cancer therapeutics 2005 4 12 1912 1922 10.1158/1535-7163.MCT-05-0184 16373706

[B29] Ances BM Letendre S Buzzell M Marquie-Beck J Lazaretto D Marcotte TD Grant I Ellis RJ Valproic acid does not affect markers of human immunodeficiency virus disease progression Journal of neurovirology 2006 12 5 403 406 10.1080/13550280600981695 17065134

[B30] Schifitto G Peterson DR Zhong J Ni H Cruttenden K Gaugh M Gendelman HE Boska M Gelbard H Valproic acid adjunctive therapy for HIV-associated cognitive impairment: a first report Neurology 2006 66 6 919 921 10.1212/01.wnl.0000204294.28189.03 16510768

[B31] Sagot-Lerolle N Lamine A Chaix ML Boufassa F Aboulker JP Costagliola D Goujard C Pallier C Delfraissy JF Lambotte O Prolonged valproic acid treatment does not reduce the size of latent HIV reservoir AIDS (London, England) 2008 22 10 1125 1129 18525257 10.1097/QAD.0b013e3282fd6ddc

