# Supplementary figures and images for: Clinical outcomes and immune benefits of anti-epileptic drug therapy in HIV/AIDS
Source: BMC Neurol. 2010 Jun 17;10:44. doi: 10.1186/1471-2377-10-44 (PMC2902446; doi:10.1186/1471-2377-10-44)

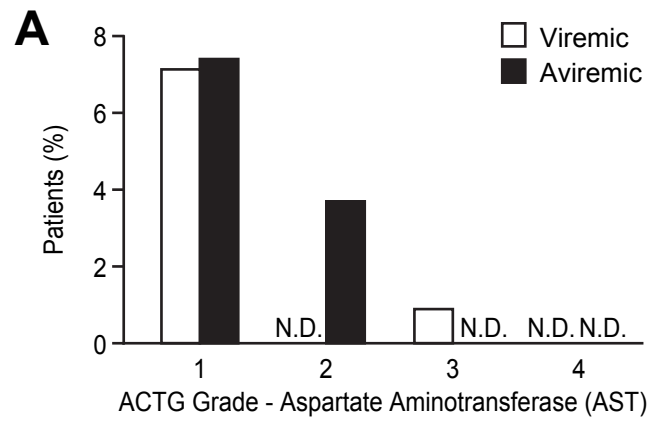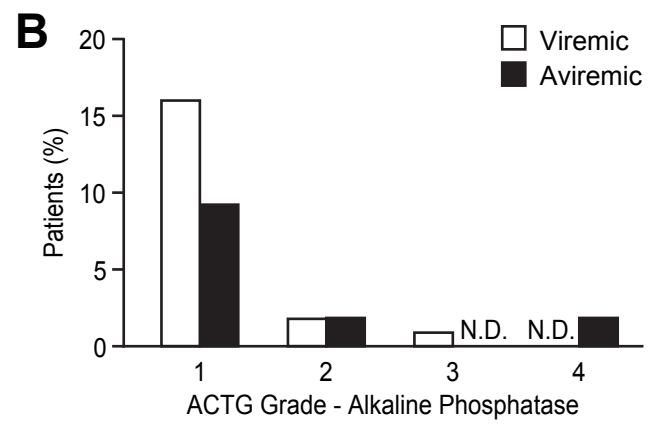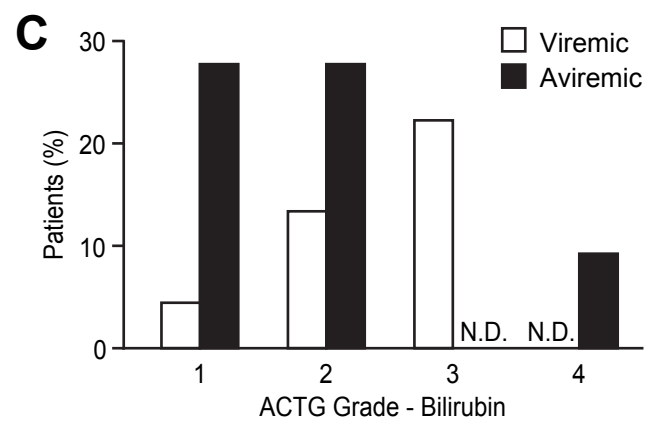

Supplement: Additional file 1 — Figure S1. Liver toxicity of AEDs. (A) and (B) Based on ACTG guidelines, aviremic patients with concurrent AED use had similar aspartate aminotransferase (AST) and alkaline phosphatise abnormalities to viremic patients (3A and 3B). In contrast, aviremic patients showed a trend toward lower hyperbilirubinemia (3E). Both aviremic and viremic patients displayed similar profile of additional LFT abnormalities after the initiation of AEDs (Figure 3D). [file 1471-2377-10-44-S1.PDF]
